# Meta-Analysis of Anti-Muscarinic Receptor Type 3 Antibodies for the Diagnosis of Sjögren Syndrome

**DOI:** 10.1371/journal.pone.0116744

**Published:** 2015-01-28

**Authors:** Chuiwen Deng, Chaojun Hu, Si Chen, Jing Li, Xiaoting Wen, Ziyan Wu, Yuan Li, Fengchun Zhang, Yongzhe Li

**Affiliations:** Department of Rheumatology and Clinical Immunology, Peking Union Medical College Hospital, Chinese Academy of Medical Sciences & Peking Union Medical College, Key Laboratory of Rheumatology and Clinical Immunology, Ministry of Education, Beijing, China; Thomas Jefferson University, UNITED STATES

## Abstract

**Purpose:**

To conduct a meta-analysis to evaluate the diagnostic value of anti-muscarinic receptor type 3 (M3R) antibodies in Sjögren syndrome (SS).

**Methods:**

Two databases, PUBMED and the Cochrane Library, were systematically searched. Approximately 2,000 participants from several studies were included in this research. STATA 11.2 software and Meta-DiSc 1.4 was used to conduct the meta-analysis.

**Results:**

Eleven studies were included in the meta-analysis. The pooled DOR was 13.00 (95% CI, 6.00–26.00). The sensitivity was 0.43 (95% CI, 0.28–0.58) and the specificity was 0.95 (95%CI, 0.91–0.97). The LR+ and LR- were 7.90 (95% CI, 4.70–13.40), 0.61 (95% CI, 0.46–0.79), respectively. The AUC was 0.89 (95% CI, 0.86–0.92).

**Conclusion:**

The anti-M3R antibody had high specificity but relatively low sensitivity for the diagnosis of SS.

## Introduction

Sjögren syndrome (SS) is a common autoimmune disease with a prevalence of 0.1% to 4.8% [1], which predominantly affects women. SS is a systemic disease, which can involve extraglandular organs, including the characteristic clinical features, dry eyes and dry mouth.

Autoantibodies usually serve as biomarkers for the diagnosis and prognosis of autoimmune diseases; in the diagnosis of SS the SSA and SSB autoantibodies play an important role. However, anti-SSA and anti-SSB antibodies are present in only 33–74% and 23–52% of SS patients, respectively [2]. In order to identify a greater proportion of cases of SS using laboratory methods, other autoantibodies, including muscarinic receptor type 3 (M3R) autoantibodies have been explored, with a view to establishing their value in the diagnosis of SS.

Muscarinic receptors are G-protein coupled acetylcholine receptors, present in plasma membranes of certain neuronal cells [3]. The anti-M3R antibody targets the M3R subtype; effects of this targeting are considered to result in elements of the pathophysiology of SS, such as impaired saliva secretion [4]. Over the past decade, numerous studies have explored the value of anti-M3R antibodies in the process of diagnosing SS. However, inconsistent conclusions relating to the diagnostic performance of anti-M3R antibodies have been drawn [5–15]. The aim of this study was to systematically review the literature to determine the diagnostic performance of anti-M3R antibodies in patients with SS.

## Methods

### Literature search

A comprehensive search of MEDLINE, EMBASE, ISI web of knowledge, and The Cochrane Library was undertaken, using the following terms; “muscarinic receptor type 3 OR M3R” and “Sjögren syndrome OR SS”. No limits were placed on ethnicity or geographic region, and all documents were updated to August 2014. Additional relevant references cited in searched articles were also selected.

### Eligibility criteria

Studies meeting the following criteria were eligible for inclusion; (1) assessed the diagnostic accuracy of testing for M3R autoantibodies in SS; (2) sufficient data reported to construct two-by-two tables; (3) testing of M3R autoantibodies by enzyme-linked immunosorbent assay; (4) there is no criteria for published language; (5) studies based on animal or cell cultures, case reports and conference abstracts without subsequent publication in full text were excluded. In the case of overlapping studies, only the study with the largest sample size was included in our analysis.

### Data extraction

Data was extracted from all selected studies by 2 independent investigators. Inter-researcher disagreements were resolved by consensus, or by a third investigator. The following data was collected from each selected study; first author’s name; publication year; country in which the study was performed; study results; detecting peptide; coupling of peptide; confirmation of peptide and diagnosis criteria. Study quality was assessed using the Quality Assessment of Diagnostic Accuracy Studies (QUADAS) tool. Authors of the identified studies were contacted via e-mail if further study details were needed.

### Statistical analysis

Statistical analysis was performed using STATA 11.2 software (Stata Corporation, College Station, TX, USA) and Meta-DiSc 1.4 (Unit of Clinical Biostatistics, Ramony Cajal Hospital, Madrid, Spain). The heterogeneity was evaluated by Cochran’s *Q*-statistic, as well as the *I^2^*-statistic; a P value >0.10 in *Q*-statistic, or *I^2^* <50% in *I^2^*-statistic, indicated lack of heterogeneity. Finally, the overall or pooled diagnostic odds ratio (DOR), sensitivity, specificity, positive likelihood ratio (LR+) and negative likelihood ratio (LR–), and their 95% CI, was obtained by a mixed-effects model, in the presence (P≤0.10 or *I^2^*>50%) or absence (P>0.10 and *I^2^*≤50%) of heterogeneity, respectively. The area under the summary receiver operating characteristic (SROC) curves represented the overall performance of the detection method. A P value <0.05 (two-sided) was considered as significant. Evaluation of multiple regression analysis and publication bias was also undertaken.

## Results

### Literature search

**Figure 1:**
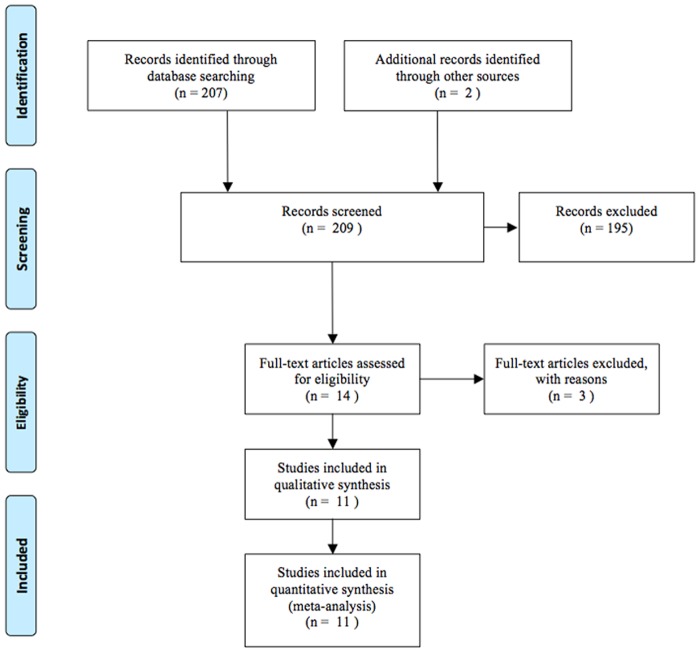
Flow chart of studies included in the meta-analysis.

Electronic and manual searches yielded a total of 69 potentially eligible articles. A flow chart of screening articles for meta-analysis is illustrated in Fig. 1. Fifty-six articles were excluded by screening the titles and abstracts. A further 4 articles were excluded following more detailed assessment (2 review articles, 1 duplicated article, 1 irrelevant article). A total of 11 eligible studies were included in the meta-analysis [5–15].

### Study characteristics

**Figure 2:**
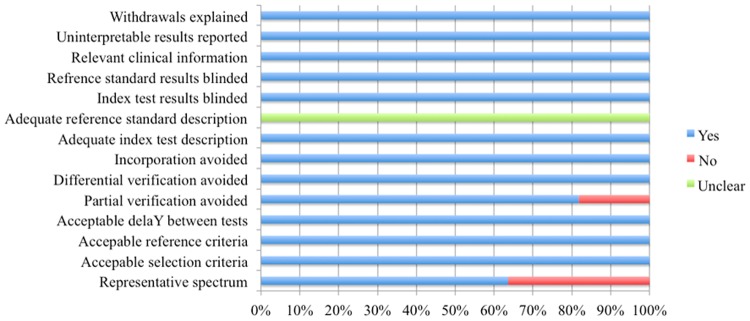
The quality assessment of included studies based on the QUADAS tool.

The characteristics of the 11 studies are summarized in Table 1. A total of 965 SS patients and 1289 controls were involved in these studies. With regards to the geographic location of the studies, 6 were carried out in Asia [6–8, 10, 11, 14], 3 in European [5, 12, 13], and 2 in America [9, 15]. Assessment using QUADAS indicated that the studies were of high quality, with positive results for at least 7/14 items (Fig. 2).

**Table 1:** Characteristics of studies included in the meta-analysis of diagnostic performance of anti-M3R antibody in SS.

**Author**	**Year**	**Region**	**TP**	**FP**	**FN**	**TN**	**Diagnosis criteria**	**Peptide used for detection**	**QUADAS Scores**
Deák et al.[5]	2012	Hungary	31	4	45	46	the 2002 American-European Consensus Criteria for SS	AGSE from the second extracellular loop of the receptor	13
			31	3	45	47		YNIP from the third extracellular loop of the receptor	
			13	4	63	46		TRIC from the third intracellular loop of the receptor	
			57	21	19	29		GST-AGSE fusion peptide	
			66	0	10	50		GST-YNIP fusion peptide	
			26	1	50	49		BSA-AGSE multiple-conjugated peptide	
He et al.[6]	2012	China	69	34	31	106	the 2002 American-European Consensus Criteria for SS	circular peptide from the second extracellular loop sequence of muscarinic receptors	13
He et al.[7]	2011	China	92	12	56	207	the 2002 American-European Consensus Criteria for SS	circular peptide from the second extracellular loop sequence of muscarinic receptors	13
			83	35	65	184		linear peptide from the second extracellular loop sequence of muscarinic receptors	
Tsuboi et al.[8]	2010	Japan	18	2	24	40	the Japanese Ministry of Health criteria for SS	peptide antigens of the N-terminal region	12
			20	3	22	39		peptide from the first extracellular loop sequence of muscarinic receptors	
			23	1	19	41		peptide from the second extracellular loop sequence of muscarinic receptors	
			19	1	23	41		peptide from the third extracellular loop sequence of muscarinic receptors	
Roescher et al.[9]	2011	America	5	2	66	35	the 2002 American-European Consensus Criteria for SS	KRAI peptide from the second extracellular loop sequence of muscarinic receptors	12
			5	1	66	36		MAP KRAI multi-antigenic peptide versions KRAI	
			4	1	67	36		Kc_c citrullinated and cyclised loop 2	
			3	1	68	36		Loop 1 peptide from the first extracellular loop sequence of muscarinic receptors	
Wu et al.[10]	2008	China	33	13	37	113	the 2002 American-European Consensus Criteria for SS	not report	13
Nakamura et al.[11]	2008	Japan	20	2	18	74	not report	peptide from the second extracellular loop sequence of muscarinic receptors	11
Kovács et al.[12]	2005	Hungary	70	31	49	126	the 2002 American-European Consensus Criteria for SS	GST-KRSE， peptide from the second extracellular loop sequence of muscarinic receptors	13
Marczinovits et al.[13]	2005	Hungary	39	0	1	40	the 2002 American-European Consensus Criteria for SS	GST-KRSE， peptide from the second extracellular loop sequence of muscarinic receptors	12
Naito et al.[14]	2005	Japan	25	4	199	326	the Japanese Ministry of Health criteria for SS	peptide from the second extracellular loop sequence of muscarinic receptors	13
Bacman et al.[15]	2001	Argentina	32	5	5	68	the 1993 European Community Criteria for SS	peptide from the second extracellular loop sequence of muscarinic receptors	12

### Meta-analysis

**Figure 3:**
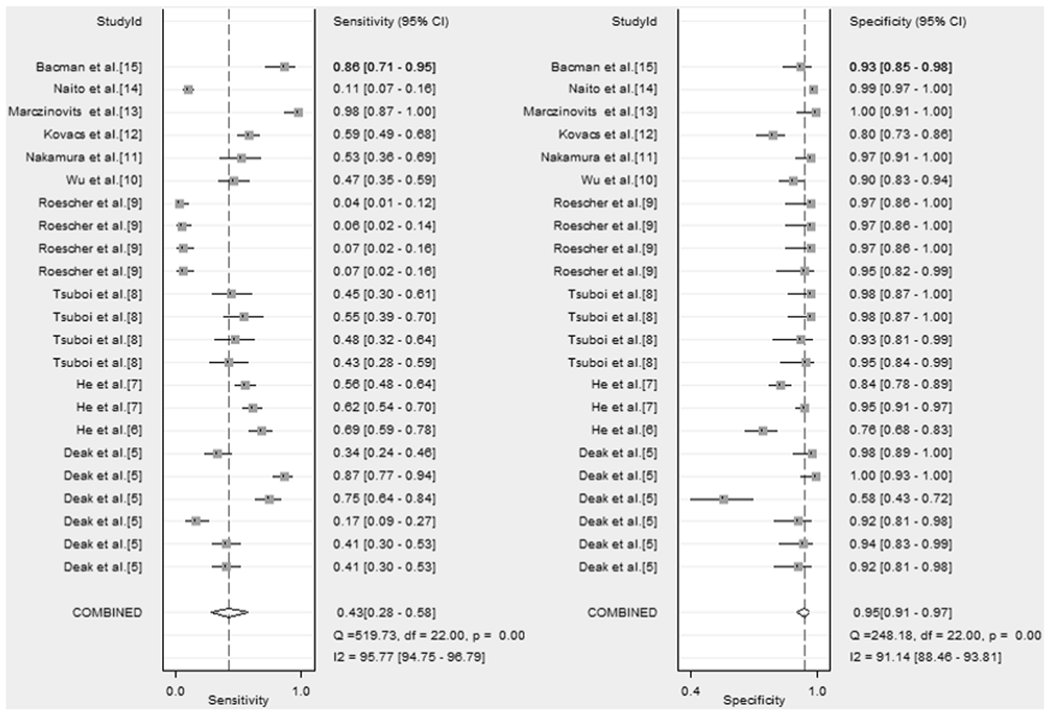
Forest plot of the accuracy of anti-M3R antibody for the diagnosis of SS.

**Figure 4:**
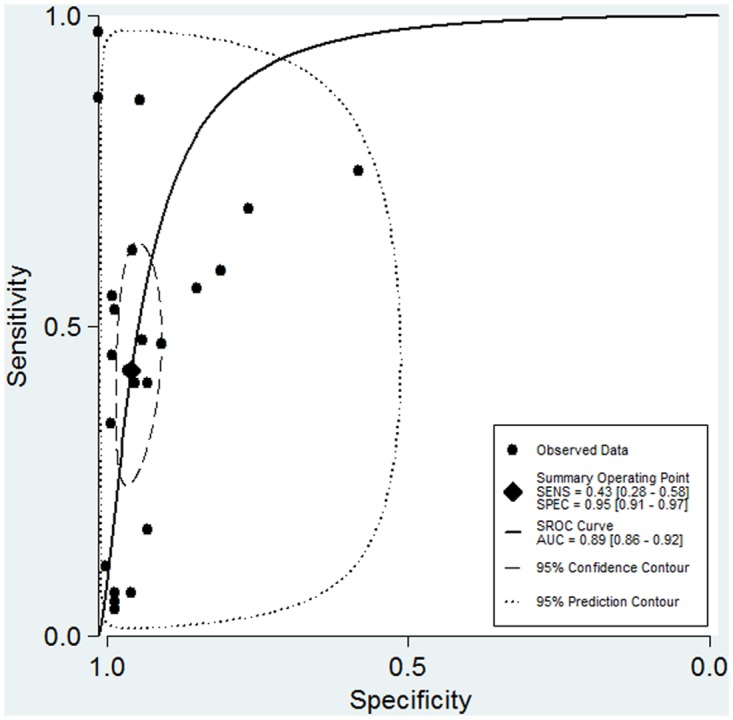
SROC of the accuracy of anti-M3R antibody for the diagnosis of SS.

The sensitivity of anti-M3R antibody testing ranged from 4%- 98%; reported specificity ranged from 58%- 100%. A mixed-effects model was used for the meta-analysis (P <0.001, *I* ^2^ = 99%). The pooled DOR was 13.00 (95% CI, 2.00–100.00). The sensitivity was 0.43 (95% CI, 0.28–0.58) and the specificity was 0.95 (95%CI, 0.91–0.97). The LR+ and LR- were 7.90 (95% CI, 4.70–13.40), 0.61 (95% CI, 0.46–0.79), respectively. The AUC was 0.89 (95% CI, 0.86–0.92). The forest plots and SROC are shown in Fig. 3 and Fig. 4, respectively.

### Multiple regression analysis and exploration for heterogeneity

**Figure 5:**
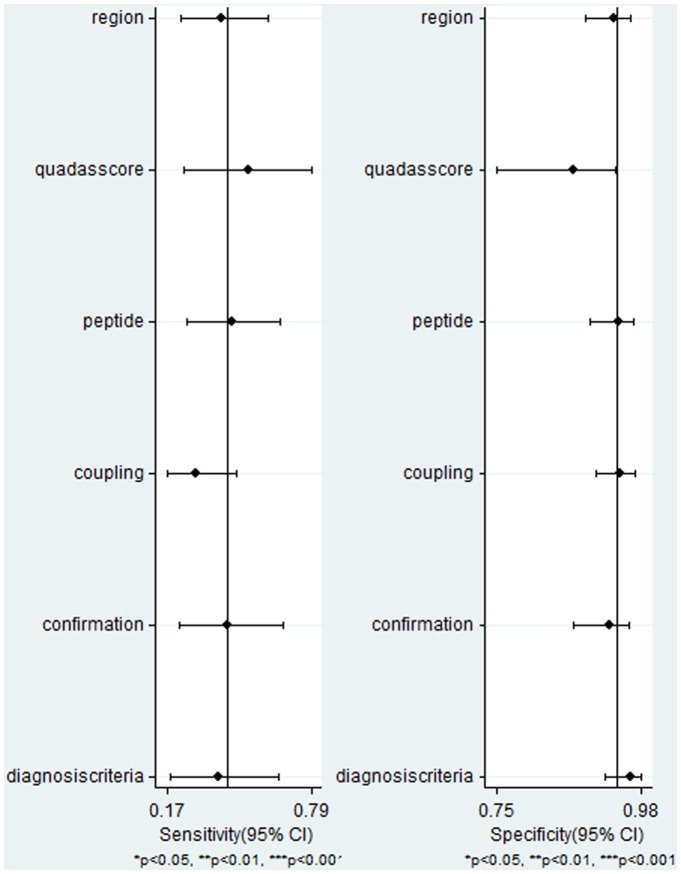
Multiple regression analysis of included studies.

Meta-regression analysis was conducted to explore possible sources of heterogeneity across the included studies. The following co-variates were evaluated as predictor variables: region; QUADAS score; peptide; coupling of peptide; confirmation of peptide; and diagnosis criteria. Results indicated that the variables we analyzed did not essentially affect the diagnostic accuracy of anti-M3R antibodies in SS (Fig. 5).

A Spearman rank correlation was also performed to confirm threshold effect; indication of threshold effect was found (Spearman correlation coefficient = 0.173, P = 0.440). In addition, the slope (b) of the regression equation did not differ from zero (P = 0.405), implying no heterogeneity among studies.

### Publication bias

**Figure 6:**
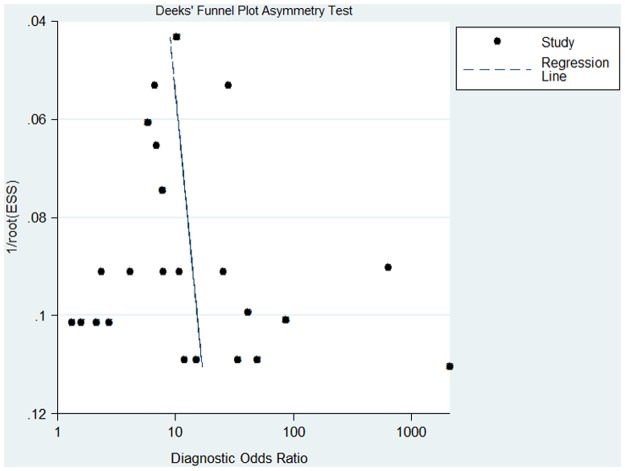
Funnel plot of included studies.

The presence of a statistically significant slope coefficient (P <0.05) was considered to indicate possible bias. We conducted funnel plots that represented a somewhat symmetric curve (Fig. 6). The slope coefficient was calculated to be 9.38±13.13, P = 0.48, indicating that no publication bias was observed in the included studies.

## Discussion

The diagnosis of SS mainly relies on the triad of clinical characteristics, results of autoantibody test results, and histological assessment. However, the common clinical manifestations, dry eyes and dry mouth, are not specific to SS. Furthermore, while histology is considered the “gold standard” for the diagnosis of SS, the invasive nature of biopsy limits its widespread application. Anti-SSA and-SSB antibody are widely accepted diagnostic biomarkers for SS; however, the diagnostic performance is not considered satisfactory, despite a high specificity, as sensitivity is relatively low [2]. Emerging autoantibodies have been explored as complementary diagnostic biomarkers, in particular anti-M3R antibodies and anti-α-fodrin antibodies. Meta-analysis has previously been performed to evaluate the diagnostic performance of anti-α-fodrin antibodies in SS; results indicated a poor sensitivity (39%) but a high specificity (83%) [16]. To date, no systemic evaluation of the anti-M3R antibody has been performed. This study is the first meta-analysis to provide precise and controlled data on the diagnostic performance of anti-M3R antibody in SS.

Anti-M3R antibody was found to be a potential diagnostic biomarker of SS, and its diagnostic performance needs to be confirmed. There were 11 eligible studies of high quality (QUADAS scores >7, Fig. 2) included in this meta-analysis (Table 1). The pooled specificity was high (0.95), in comparison with a relatively low pooled sensitivity (0.43), which revealed that the diagnostic performance of anti-M3R antibody could identify controls better than anti-α-fodrin antibodies. The SROC curve implies anti-M3R antibodies showed moderate diagnostic performance for SS. The pattern of the points in the SROC curve did not suggest a ‘shoulder-arm’ shape, and the AUC of SROC was 0.89 (95% CI, 0.86–0.92). Taken together, these results indicate that anti-M3R antibody had a modest level of overall diagnostic accuracy for SS.

In order to explore the heterogeneity found across studies (P<0.001, *I* ^2^ = 99%), meta-regression analysis and Spearman rank correlation were performed. We considered these variables might lead to the heterogeneity: Firstly, it’s commonly recognized that different part of the M3R peptide, or M3R peptide coupling with or without larger molecular, or different modification of confirmation (linear/circular) have diverse antigenicity. Thus, we summarized the peptide that used in the detection of anti-M3R antibody (Table 1). Results showed that most studies used synthetic linear peptide from the second extracellular loop of the M3R, no heterogeneity was found between the researches that used different peptides; Secondly, previous research reported that there is different incidence of SS between Chinese and Japanese, however, we didn’t found heterogeneity among all the regions; Thirdly, different diagnosis criteria were applied among these studies, which might lead to the heterogeneity of included patients and eventually affected the diagnosis accuracy of anti-M3R antibody. Also no statistical heterogeneity was found. The last but not the least, different QUADAS score of the included studies did not influence the diagnostic accuracy of anti-M3R antibodies.

Some limitations in this meta-analysis need to be noted. Since not all the included studies provided clinical information, we did not evaluate the effect of different clinical manifestation of the disease, which may introduce analysis bias. In addition, we did not compare the diagnostic accuracy of anti-M3R antibody with the accuracy of anti-SSA and-SSB antibodies for the lack of researches.

In conclusion, anti-M3R antibodies are a modestly effective diagnostic biomarker for SS, with a low sensitivity but a high specificity. Combing anti-SSA, -SSB, and -M3R antibody assays may enhance diagnostic performance, and decrease the misdiagnosis rate.

## Supporting Information

S1 PRISMA Checklist (DOC)
